# First 100 Reported Cases of Supine Percutaneous Nephrolithotomy in Malaysia: An Alternative Effective and Safe Approach to Treat Renal Stones

**DOI:** 10.21315/mjms2024.31.3.9

**Published:** 2024-06-27

**Authors:** Han Kun Ng, Chee Hoong Loo, Meng Shi Lim

**Affiliations:** Department of Urology, Sarawak Heart Centre, Sarawak, Malaysia

**Keywords:** supine percutaneous nephrolithotomy, renal stone, urolithiasis, endourology

## Abstract

**Background:**

Supine percutaneous nephrolithotomy (s-PCNL) offers great benefits from urological and anaesthetic points of view. We present the first evaluation of the outcomes of s-PCNL in Malaysia. Our aim was to explore the safety and efficacy of s-PCNL.

**Methods:**

Institutional review board approval was obtained from the National Medical Research Register (NMRR ID-21002225-WLP). We retrospectively reviewed 115 patients with renal pelvis stones who underwent single renal access during s-PCNL between November 2020 and May 2023. Patients who underwent simultaneous ipsilateral or contralateral endourological procedures were included. The data were analysed to determine stone-free rates (SFR), major complication rates, blood transfusion rates, operative times and lengths of hospital stay (LOS).

**Results:**

The SFR was higher for the single middle calyceal renal access (MCA) group than for the lower calyceal renal access (LCA) or upper calyceal renal access (UCA) groups (OR: 1.76; 95% confidence interval [CI]: 0.63, 4.92). In total, 0, 1 and 2 patients had major complications in the UCA, MCA and LCA groups, respectively (*P* = 0.453). One of the 115 patients (0.9%) needed blood transfusion. Subgroup analysis revealed mean operative times of 76.3 min and 78.6 min for patients who underwent sole s-PCNL (PCNL-only group) and those who had simultaneous ipsilateral and contralateral endourological procedures (PCNL-plus group), respectively (*P* = 0.786). The overall mean LOS was 2.9 days.

**Conclusion:**

s-PCNL is a safe and effective alternative treatment for renal stones. We would recommend s-PCNL for patients who require an ipsilateral/contralateral endourological procedure (URS/RIRS) because it is time-efficient. All renal accesses are safe. Single MCA is recommended for complete stone clearance.

## Introduction

Percutaneous nephrolithotomy (PCNL) is strongly recommended for the treatment of large, multiple and complex renal stones (1, 2). It has the highest single-treatment stone-free rate (SFR) when compared with other renal stone treatment modalities, such as extracorporeal shockwave lithotripsy (ESWL) (3) and retrograde intrarenal surgery (RIRS) (4). The main concern of PCNL is its higher rate and severity of complications compared to other treatment options (5).

Percutaneous renal access is the most important step in PCNL because it is the only way to reach and manage stones. This access can be made through the upper, middle or lower calyx. Many urologists prefer lower calyceal renal access (LCA) because of the lower risk of pleural injury (6); however, the upper calyceal renal access (UCA) is preferred for the treatment of complex and staghorn stones (7, 8). By contrast, middle calyceal renal access (MCA) is often underutilised in standard PCNL (6, 9).

From the urologist’s point of view, supine PCNL (s-PCNL) offers better ergonomics for the surgeon, shorter operative time due to easier patient positioning, reduced intrarenal pressure (and a consequent reduced risk of urosepsis) and room for endoscopic combined intrarenal surgery (ECIRS) or simultaneous bilateral endoscopic surgery (SBES).

In addition, s-PCNL offers clear advantages over prone PCNL in terms of anaesthesiologist management. This includes improved access to the patient for cardiovascular and pulmonary management (especially during an emergency situation), less risk of injury to the central and peripheral nervous system, less risk of thromboembolism due to the lack of inferior vena cava compression (10) and improved ventilator-associated parameters for obese patients (11). Moreover, no extra anaesthesiologic equipment is needed (e.g. reinforced endotracheal tubes, stabilising helmets or specialised paddings, which may add additional cost to the procedure).

The present study was conducted to compare the efficacy and safety of a single UCA, MCA or LCA in s-PCNL for the treatment of renal pelvis stones.

## Methods

### Patient Selection

This retrospective cohort study sampled the subjects using a convenience sampling method. We performed s-PCNL on 134 patients at the Sarawak Heart Institute from November 2020 to May 2023. Patients with a renal pelvic stone (with or without calyceal extension) and a maximum stone diameter ranging from 2 cm to 3 cm and who were treated with a single renal access were included in the study. We also included patients who underwent a simultaneous ipsilateral or contralateral endourological procedure (e.g. vesicolithotripsy, ureterorenoscopy) for a renal/ureteric stone with a maximum diameter of less than 1 cm. Patients with staghorn calculi, a pre-existing nephrostomy tube or radiolucent stones were excluded.

### Methodology

Computed tomography (CT) was used for preoperative evaluation of the stone burden, pelvicalyceal system anatomy and retrorenal colon. Laboratory tests included urine analysis and culture, serum creatinine, full blood count, C-reactive protein and coagulation profile. Patients with coagulopathy or receiving anticoagulants did not undergo s-PCNL. Culture-specific antibiotics were administered to patients with infected urine cultures and the s-PCNL was performed when the cultures no longer showed infection.

All patients provided informed consent before their operations. The operations were performed by a single urologist in the centre. After administration of prophylactic antibiotics (Cefoperazone 1 g intravenously) and under general anaesthesia, the patient was placed in a modified Giusti position and the posterior axillary line was marked as the anterior limit of skin puncture during PCNL. The patient was then cleaned and draped, followed by placement of a ureteric catheter at the renal pelvis under fluoroscopic guidance. A retrograde renal pyelogram (RPG) was then performed to delineate the pelvicalyceal system to decide on the most suitable calyx for percutaneous renal access. For example, a focal caliectasis is preferred over a non-caliectasis calyx. Most frequently, a MCA is feasible, as it has the easiest axis puncture unless there is no middle calyx in the renal units.

Percutaneous renal access to the most suitable calyx was achieved either under bi-plane C-arm fluoroscopic guidance, USG guidance or both methods for complete clearance of the renal stones. After passing a 0.035 inch Roadrunner guidewire into the pelvicalyceal system or ureter, the tract was dilated using sequential dilators (Amplatz) to the size of a 24Fr sheath. A standard rigid nephroscope (22Fr) was used for a 24Fr tract. A continuous irrigation system was connected to the nephroscope with the irrigation saline at a height of 60 cm above the patient’s centre point. The stones were disintegrated with ultrasonic lithotripters. Large fragments were evacuated through the sheath by the application of a vacuum cleaner effect or with forceps. The collecting system was inspected for residual stones using a rigid nephroscope and/or a 16Fr-flexible cystoscope. In addition, fluoroscopy was used to ensure complete clearance of all stone fragments. At the end of the procedure, an 18Fr nephrostomy tube was inserted, with or without a ureteric stent, under fluoroscopic guidance. A Foley urethral catheter was then inserted.

Another urologist performed a simultaneous endourological procedure, if needed. In the case of urinary bladder stones of size ≤ 1 cm, we routinely use Maumayer stone forceps for treatment. In the case of ipsilateral/contralateral ureteric stones of size ≤ 1 cm, a 6.5Fr/7.5Fr ureteroscope (URS) and a Ho:YAG laser lithotriptor were used. In the case of contralateral renal stones of size ≤ 1cm, we routinely inserted a ureteral access sheath (11/13Fr), followed by a 9.5Fr flexible URS (RIRS) and then fragmented the stone using a Ho:YAG laser lithotriptor. A ureteric stent was placed for complicated URS/RIRS.

A post-operative full blood count was taken to assess any drop in haemoglobin as a surrogate for the degree of intra-operative haemorrhage. A plain film of the kidney, ureter and bladder (KUBXR) was obtained on the first post-operative day to confirm the proper stent position (if any). If no complications were evident and the urine was clear, the urethral catheter, ureteric catheter and nephrostomy were removed, and the patient was discharged. The stent (if any) was removed at 2 weeks post-operatively under local anaesthesia.

On follow-up, a KUBXR was performed 30 days later to evaluate stone-free status. CT scans for reassessment of stone clearance were reserved only for radiolucent stone cases due to financial constraints and those patients were not included in this study. Patients who were ‘stone-free’ were followed up 6-monthly for stone recurrence, whereas patients who were not ‘stone-free’ were counselled for further intervention (i.e. URS, RIRS, ESWL or a re-do of the PCNL).

### Measures

Demographics and post-operative outcomes were recorded. Post-operative outcomes included operative time, length of hospital stays (LOS), SFR, major complication rate and transfusion rate. Major complications in our study were defined as Clavien-Dindo Class 3 and above. The stone clearance status was assessed by KUBXR at 1 month post-operatively and the stone-free state referred to a patient who might still have a residual stone of maximum diameter less than 4 mm (12).

### Sample Size Statement

Our study needed 90 or more patients to have a confidence level of 95% so that the real value is within ±5% of the measured value, according to the data from Falahatkar et al. (13). The significance level was set at 0.05. Therefore, after estimating a 20% dropout, at least 110 patients were needed to detect an effect at 80% power.

### Statistical Analysis

The mean and standard deviation (SD) or the median and interquartile range (IQR) were used for the descriptions of quantitative variables, whereas frequency and percentage were used for qualitative variables. The SFR, major complication rate, transfusion rate, mean operative time and LOS were analysed based on three groups: i) a single UCA group, ii) a single MCA group and iii) a single LCA group. A subgroup analysis of operative time was conducted for stone-free cases by grouping them into patients who underwent solely s-PCNL (PCNL-only group) and patients who underwent simultaneous ipsilateral/contralateral endourological (PCNL-plus group) procedures. Continuous variables were compared using the independent *t*-test (two groups) and one-way ANOVA test (more than two groups), as appropriate. Categorical variables were compared using the chi-square test. A *P*-value < 0.05 was considered statistically significant. Statistical analyses were conducted using IBM SPSS version 25.0, for the Macintosh OS.

## Results

The patients’ demographic data are presented in Table 1. In total, 115 patients were recruited, including 30, 43 and 42 patients in the UCA, MCA and LCA groups, respectively. Most of the patients (at least 95%) were in the American Society of Anaesthesiologists (ASA) Class 1 to 2 and about one-third of patients were obese in all study groups. No statistically significant differences were detected among the patient groups with respect to age, gender, ASA class, BMI, stone maximum diameter, stone density (Hounsfield unit [HU]) or stone laterality. None of our patients had a kidney abnormality (i.e. horseshoe kidney, duplex system, malrotation, etc.). The median follow-up was 13.2 months at the date of writing (IQR 7.5 months–15.4 months).

The operative details and post-operative outcomes are summarised in Table 2. Efficacy is elaborated in terms of SFR, operative time and LOS, whereas safety is assessed based on major complication rate, haemoglobin drop and transfusion rate.

### Stone-Free Rate

Overall, 92 patients (80%) were stone free after s-PCNL. The SFR was higher in the MCA group than in the LCA and UCA groups (OR: 1.76; 95% CI: 0.63, 4.92)

### Operative Time

Mean operative times for the UCA, MCA and LCA groups were 81.6 min, 88.6 min and 84.1 min, respectively (*P* = 0.707). Subgroup analysis of the PCNL-only and PCNL-plus groups revealed no statistical difference in the mean operative time, which were 76.3 min and 78.6 min, respectively (*P* = 0.786).

### Length of Hospital Stay

The overall mean LOS was 2.9 days.

### Major Complication Rate

The overall major complication rate was 2.6% (three patients). There were 0/1/2 patients who had major complication in UCA/MCA/LCA group (*P* = 0.453). One patient in the MCA group required selective renal angioembolisation under local anaesthesia for renal pseudoaneurysm (Clavien 3A). In the LCA group, one patient with an abdominal compartment syndrome complication post-PCNL, likely due to irrigant extravasation, required a short ICU stay (Clavien 4A) and one patient with an obstructed migrated stent in a solitary kidney required a stent change (Clavien 3A). There were no events of pleural injury, colonic injury, splenic injury or death in our study.

### Haemoglobin Drop and Transfusion Rate

The mean haemoglobin drop was 1.35g/dL. One out of 115 patients (0.9%) required a blood transfusion, which was in the MCA group.

## Discussion

Our study reports data from an urology department in the Sarawak Heart Centre, a district hospital in Malaysia. The department is run by a consultant urologist, a trainee urologist and a medical officer. Only one or two anaesthesiologists are comfortable ventilating patients in the prone position; therefore, we are one of very few centres that perform s-PCNL on a regular basis. Based on our literature review, no one has yet reported the outcomes of s-PCNL in Malaysia.

Undoubtedly, renal access in PCNL plays an important role in the success of surgery. Traditionally, the UCA and LCA are the most preferred because, theoretically, they follow the natural longitudinal axis of the kidney and assume that a stone can be cleared in a single access. However, unlike the case for prone PCNL, the upper pole calyx of a kidney in the modified Giusti position is not readily accessible, as the kidney is surrounded by the liver (right side) and the spleen or pleura (left side). Therefore, to access the upper pole in s-PCNL, a skilful urologist is required to tilt the kidney inferiorly using a Chiba needle under fluoroscopic guidance before puncture. Although no vital solid organs are present in the vicinity of a puncture in the lower pole of a kidney, access to the lower pole renal calyx can be difficult, especially during tract dilatation, due to its high mobility and because it almost always ends with a long tract. Conversely, in our opinion, the middle calyx renal access in s-PCNL is easy, as it is always subcostal and it has a shorter skin–calyceal distance when compared to UCA or LCA. Furthermore, the posterior middle calyx is easy to identify when obtaining renal access using ultrasound guidance compared with fluoroscopic guidance. From our early experience with s-PCNL, we also realised that manoeuvring the nephroscope is always limited by the costal margin (in upper pole access) and the iliac crest (in lower pole access), thus limiting access to the minor calyx with an extreme angle. Moving a nephroscope via a LCA becomes more difficult when a long tract is created.

With better nephroscope mobility within the pelvicalyceal system, the SFR, not surprisingly, was higher in the single MCA group than in the single UCA/LCA group. Yan Song et al. (14) also compared the SFR between different calyx access groups and reported a significantly higher SFR for that single MCA than for single UCA/LCA (98.2% versus 93.3/84.3%; *P* = 0.037). A study by Falahatkar et al. (13), which excluded upper pole renal stones and upper pole calyceal access renal units, showed that the SFR was higher for a single MCA than for a single LCA (89.6% versus 76.2%; *P* = 0.054). The higher SFR may result from the easy access via the middle calyx, a proper angle between the middle calyx tract and long axis of the kidney, optimal alignment of this access with the ureteropelvic junction and easy access to the renal pelvis and upper ureter for stone removal.

In terms of the safety profile, our study showed a lower complication rate compared to other s-PCNL studies. For example, Yan Song et al. (14) reported complication rates of 17.8%, 14% and 15.7% for their UCA, MCA and LCA groups, respectively (*P* = 0.862). Falahatkar et al. (13) also pointed out that MCA had an acceptable complication rate (10.4%) when compared to LCA (14.8%) (*P* = 0.4). Boon et al. (15) found that the risk of injuring the colon increased when puncturing the lower pole of kidneys by fluoroscopy. We think that the use of ultrasound during renal access (with or without fluoroscopy) may play a role in reducing the complication rate. This is because real-time ultrasound guidance allows assessment of the depth of the puncture to prevent counter-puncture; therefore, one can avoid injuring the lung or colon and improve the rate of trans-papillary renal puncture, which is well known to generate less bleeding (16).

The mean operative time in our study was comparable between the three groups. However, based on our subgroup analysis of 92 patients who had stone-free status, no extra time was needed for the simultaneous ipsilateral/contralateral endoscopic procedure for stone sizes under 1 cm in size (e.g. vesicolithotripsy, URS and contralateral RIRS). This was because the modified Giusti position allows simultaneous percutaneous renal and urethral access. In prone PCNL, the patient is first placed in the lithotomy position for retrograde insertion of the ureteric catheter, followed by the prone position. Hence, extra time and manpower are needed for the prone PCNL procedure. Furthermore, prone PCNL also hinders retrograde ureteric access for simultaneous ipsilateral/contralateral URS/RIRS. We have to emphasise that the use of s-PCNL for the treatment of renal stones is especially beneficial for the public urological service in Malaysia because it is time- and manpower-efficient.

## Conclusion

s-PCNL is a safe and effective alternative treatment for renal stones. We would highly recommend s-PCNL for the treatment of patients with renal stones who also need an ipsilateral/contralateral endourological procedure (Vesicolithotripsy/URS/RIRS). This is especially the case in the public urological service in Malaysia, where the patient load is high. All percutaneous renal accesses in s-PCNL are generally safe. The single MCA is recommended for maximum stone clearance.

### Study Limitations

This study had some limitations. One was that all the cases were performed by a single consultant urologist; therefore, the results may not be reproducible by others. Second, this is a retrospective analysis, so the collection of data may be incomplete, especially if the patients come from far-away locations in Sarawak (e.g. Lawas, Limbang, Kapit and Belaga). Third, the number of subjects was relatively small when compared to prone PCNL subjects because s-PCNL is not as popular as prone PCNL in Malaysia. Furthermore, evaluation of the ‘stone-free’ status post-operatively might be inadequate with KUBXR due to resource limitations in a district hospital. Nevertheless, all of the patients selected for this study had a radiopaque renal stone and KUBXR alone is a reliable tool for following-up on stone-free status. Lastly, although s-PCNL is a good approach, formal training is needed to reduce the learning curve of newly inducted urologists.

## Figures and Tables

**Table 1:** Patient and stone characteristics

Characteristics	UCA (*n* = 30)	MCA (*n* = 43)	LCA (*n* = 42)	*P*-value
Age: mean (SD) years old	48.5 (14.1)	46.9 (10.4)	51.7 (11.6)	0.176
Gender: *n* (%)				0.316
Male	19 (63.3)	23 (53.5)	19 (45.2)	
Female	11 (36.7)	20 (46.5)	23 (54.8)	
ASA class: *n* (%)				0.757
1–2	29 (96.7)	41 (95.3)	39 (92.9)	
3–4	1 (3.3)	2 (4.7)	3 (7.1)	
BMI: *n* (%) kg/m^2^				0.148
< 30	18 (60.0)	31 (72.1)	34 (81.0)	
≥ 30	12 (40.0)	12 (27.9)	8 (19.0)	
Stone laterality: *n* (%)				0.351
Right	13 (43.3)	26 (60.5)	22 (52.4)	
Left	17 (56.7)	17 (39.5)	20 (47.6)	
Maximum stone diameter: mean (SD) cm	2.6 (0.5)	2.9 (0.8)	2.8 (0.7)	0.574
HU: mean (SD)	1212.5 (317.5)	1116.0 (272.8)	1221.8 (229.4)	0.164

Note: UCA = upper calyceal renal access; MCA = middle calyceal renal access; LCA = lower calyceal renal access; ASA = American Society of Anaesthesiologists; BMI = body mass index; HU = Hounsfield unit

**Table 2:** Operative details and post-operative outcomes

Parameters	UCA (*n* = 30)	MCA (*n* = 43)	LCA (*n* = 42)	*P*-value
Stone free: *n* (%)				0.397
Yes	22 (73.3)	37 (86.0)	34 (81.0)	
No	8 (26.7)	6 (14.0)	8 (19.0)	
Major complication: *n* (%)				0.453
Yes	0 (0.0)	1 (2.3)	2 (4.8)	
No	30 (100.0)	42 (97.7)	40 (95.2)	
Haemoglobin drop: mean (SD) g/dL	1.1 (0.4)	1.3 (0.9)	1.2 (0.5)	0.543
Transfusion needed: *n* (%)				0.430
Yes	0 (0.0)	1 (2.3)	0 (0.0)	
No	30 (100.0)	42 (97.7)	42 (100.0)	
LOS: mean (SD) day	2.9 (2.5)	2.6 (2.8)	2.4 (1.7)	0.171
Operative time: mean (SD) min	81.6 (36.1)	88.6 (39.6)	84.1 (32.6)	0.707

Note: UCA = upper calyceal renal access; MCA = middle calyceal renal access; LCA = lower calyceal renal access; LOS = length of hospital stay
